# Finding Pairwise Intersections Inside a Query Range

**DOI:** 10.1007/s00453-017-0384-3

**Published:** 2017-10-31

**Authors:** Mark de Berg, Joachim Gudmundsson, Ali D. Mehrabi

**Affiliations:** 1 Department of Computer Science, TU Eindhoven, Eindhoven, The Netherlands; 2 School of IT, University of Sydney, Sydney, Australia

**Keywords:** Data structures, Computational geometry, Intersection searching

## Abstract

We study the following problem: preprocess a set $\mathcal {O}$ of objects into a data structure that allows us to efficiently report all pairs of objects from $\mathcal {O}$ that intersect inside an axis-aligned query range ${Q}$. We present data structures of size $O(n\cdot {{\mathrm{polylog\,}}}n)$ and with query time $O((k+1)\cdot {{\mathrm{polylog\,}}}n)$ time, where *k* is the number of reported pairs, for two classes of objects in ${\mathbb R}^2$: axis-aligned rectangles and objects with small union complexity. For the 3-dimensional case where the objects and the query range are axis-aligned boxes in ${\mathbb R}^3$, we present a data structure of size $O(n\sqrt{n}\cdot {{\mathrm{polylog\,}}}n)$ and query time $O((\sqrt{n}+k)\cdot {{\mathrm{polylog\,}}}n)$. When the objects and query are fat, we obtain $O((k+1)\cdot {{\mathrm{polylog\,}}}n)$ query time using $O(n\cdot {{\mathrm{polylog\,}}}n)$ storage.

## Introduction

The study of geometric data structures is an important subarea within computational geometry, and range searching forms one of the most widely studied topics within this area [4, 15]. In a range-searching query, the goal is to report or count all points from a given set $\mathcal {O}$ that lie inside a query range ${Q}$. The more general version, where $\mathcal {O}$ contains other objects than just points and the goal is to report all objects intersecting ${Q}$, is often called intersection searching and it has been studied extensively as well. A common characteristic of almost all range-searching and intersection-searching problems studied so far, is that whether an object $o_i\in \mathcal {O}$ should be reported (or counted) depends only on $o_i$ and ${Q}$. In this paper we study a range-searching variant where we are interested in reporting *pairs* of objects that satisfy a certain criterion. In particular, we want to preprocess a set $\mathcal {O}=\{o_1,\ldots ,o_n\}$ of *n* objects in ${\mathbb R}^2$ or ${\mathbb R}^3$ such that, given a query range ${Q}$, we can efficiently report all pairs of objects $o_i,o_j$ that intersect inside ${Q}$.

Our motivation for studying these problems is the following. Suppose we are given a collection of *n* discrete trajectories representing the movements of, say, people. Each trajectory is a sequence of locations (points in ${\mathbb R}^2$) with a corresponding time stamp; for discrete trajectories the movement in between consecutive locations is not considered. The query we are interested in is: which pairs of people met inside a given rectangular query region ${Q}$? A natural way to define that two people meet is to require that they are within a given distance *D* from each other. When we restrict our attention to a fixed time instance, we can place a disk of radius *D* / 2 around the location of each person and the question becomes: which pairs of disks intersect within ${Q}$? When we consider the $\ell _{\infty }$ metric, we get the same problem but now for squares instead of disks. A more general version of the query also specifies a time interval *I*: which pairs of people met within a region ${Q}'$ during time interval *I*? To deal with the fact that the time stamps may not be synchronized for the different trajectories, we assume that each location is valid for some interval of time. If we then model time as the third dimension and consider distances in the $\ell _{\infty }$ metric, we get the question: which pairs of boxes (which are the product of a square around a location and a time interval) intersect with the query box ${Q}:= {Q}'\times I$?

An obvious approach to our problem is to precompute all intersections between the objects and store the intersections in a suitable intersection-searching data structure. This may give fast query times, but in the worst case any two objects intersect, so $\Omega (n^2)$ is a lower bound on the storage for this approach. The main question is thus: can we achieve fast query times with a data structure that uses subquadratic (and preferably near-linear) storage in the worst case?

Rahul et al. [21] answered this question affirmatively when ${Q}$ is an axis-aligned rectangle in ${\mathbb R}^2$ and the objects are axis-aligned line segments. Their data structure uses $O(n\log n)$ storage and answers queries in time $O(\log n + k)$, where *k* is the number of answers. Our contribution is to obtain similar results for a broader class of objects than those of [21], namely axis-aligned rectangles and objects with small union complexity. For axis-aligned rectangles our data structure uses $O(n\log n)$ storage and has $O(\log n\log ^* n + k\log n)$ query time,^1^ where *k* is the number of reported pairs of objects. Our data structure for classes of objects with small union complexity—disks and other types of fat objects are examples—uses $O(U(n)\log n)$ storage, where *U*(*n*) is maximum union complexity of *n* objects from the given class, and it has $O((k+1)\log ^2 n)$ query time. We also consider a 3-dimensional version of the problem, where the range ${Q}$ and the objects in $\mathcal {O}$ are axis-aligned boxes. Here our data structure uses $O(n\sqrt{n}\log n)$ storage and $O((\sqrt{n}+k)\log ^2 n)$ query time. When the query range and the objects are fat, we improve this to $O(n\log ^2 n)$ storage and $O((k+1)\log ^2 n)$ query time.

*Related work* The paper by Rahul et al. [21] mentioned above studies the same problem as we do (in a less general setting). There are a few more papers dealing with related problems. Das et al. [10] have studied the problem of preprocessing a set *H* of *n* horizontal and *V* of *n* vertical segments in the plane into a data structure such that given an axis-parallel query rectangle ${Q}$ and a parameter $\delta$, all the triples (*h*, *v*, *p*) where $h\in H$, $v\in V$, and *p* is an endpoint of either of the segments and $h\cap v\cap {Q}\ne \emptyset$ and $dist(h\cap v,p)\leqslant \delta$ can be reported efficiently. Their data structure needs $O(n\log ^3 n)$ space and is able to answer the desired queries in $O(\log ^2 n+\#\text{ answers })$ time. Abam et al. [1], Gupta [16], and Gupta et al. [17] have presented data structures that return the closest pair inside a query range.

## Axis-Aligned Objects

In this section we study the case where the set $\mathcal {O}$ is a set of *n* axis-aligned rectangles in ${\mathbb R}^2$ or boxes in ${\mathbb R}^3$. We assume throughout the paper that the objects in $\mathcal {O}$ as well as the query rectangles are closed sets. Our approach for these cases is the same and uses the following two-step query process.

1. Compute a *seed set* $\mathcal {O}^*({Q})\subseteq \mathcal {O}$ of objects such that the following holds: for any two objects $o_i,o_j$ in $\mathcal {O}$ such that $o_i$ and $o_j$ intersect inside ${Q}$, at least one of $o_i,o_j$ is in $\mathcal {O}^*({Q})$.
2. For each seed object $o_i\in \mathcal {O}^*({Q})$, perform an intersection query with the range $o_i\cap {Q}$ in the set $\mathcal {O}$, to find all objects $o_j\ne o_i$ intersecting $o_i$ inside ${Q}$.

For this approach to be efficient, $\mathcal {O}^*({Q})$ should not contain too many objects that do not give an answer in Step 2. For the planar case we will ensure $|\mathcal {O}^*({Q})| =O(1+k)$, where *k* is the number of pairs of objects intersecting inside ${Q}$, while for the 3-dimensional case we will have $|\mathcal {O}^*({Q})| =O(\sqrt{n}+k)$.

### The Planar Case

**Fig. 1:**
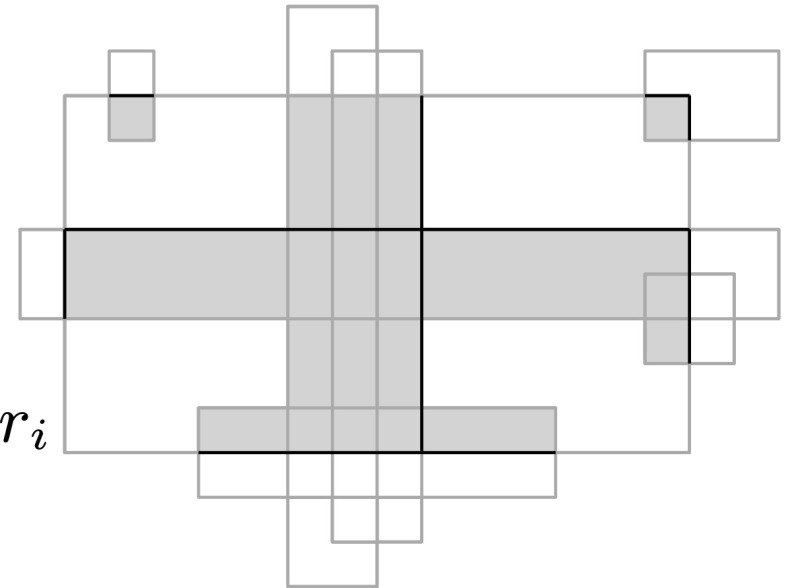
Gray areas are intersections of other rectangles with $r_i$ black segments indicate witness segments

Let $\mathcal {O}=\{r_1,\ldots ,r_n\}$ be a set of axis-aligned rectangles in ${\mathbb R}^2$. The key to our approach is to be able to efficiently find the seed set $\mathcal {O}^*({Q})$. To this end, during the preprocessing we compute a set *W* of axis-aligned *witness segments*. For each rectangle $r_i\in \mathcal {O}$ we define at most ten witness segments, two for each edge of $r_i$ and two in the interior of $r_i$, as follows—see also Fig. 1.

Let *e* be an edge of $r_i$, and consider the set $S(e) := e \cap \left( \cup _{j\ne i} r_j\right)$, that is, the part of *e* covered by the other rectangles. The set *S*(*e*) consists of a number of sub-edges of *e*. If *e* is vertical then we add the topmost and bottommost sub-edge from *S*(*e*) (if any) to *W*; if *e* is horizontal we add the leftmost and rightmost sub-edge to *W*. The two witness segments in the interior of $r_i$ are defined as follows. Suppose there are vertical edges (belonging to other rectangles $r_j$) completely crossing $r_i$ from top to bottom. Then we put $e'\cap r_i$ into *W*, where $e'$ is the rightmost such crossing edge. Similarly, we put into *W* the topmost horizontal edge $e''$ completely crossing $r_i$ from left to right. Our data structure to find the seed set $\mathcal {O}^*({Q})$ now consists of the following components.

- We store the witness set *W* in a data structure $\mathcal {D}_1$ that allows us to report the witness segments that intersect the query rectangle ${Q}$.
- We store the vertical edges of the rectangles in $\mathcal {O}$ in a data structure $\mathcal {D}_2$ that allows us to decide if the set ${\textsf {V}}({Q})$ of edges that completely cross a query rectangle ${Q}$ from top to bottom, is non-empty. The data structure should also be able to report all (rectangles corresponding to) the edges in ${\textsf {V}}({Q})$.
- We store the horizontal edges of the rectangles in $\mathcal {O}$ in a data structure $\mathcal {D}_3$ that allows us to decide if the set $\textsf {H}({Q})$ of edges that completely cross a query rectangle ${Q}$ from left to right, is non-empty.
- We store the set $\mathcal {O}$ in a data structure $\mathcal {D}_4$ that allows us to report the rectangles that contain a query point *q*.
  Step 1 of the query procedure, where we compute $\mathcal {O}^*({Q})$, proceeds as follows.
  1. Perform a query in $\mathcal {D}_1$ to find all witness segments intersecting ${Q}$. For each reported witness segment, insert the corresponding rectangle into $\mathcal {O}^*({Q})$.
  2. Perform queries in $\mathcal {D}_2$ and $\mathcal {D}_3$ to decide if the sets ${\textsf {V}}({Q})$ and $\textsf {H}({Q})$ are both non-empty. If so, report all rectangles corresponding to edges in ${\textsf {V}}({Q})$ and put them into $\mathcal {O}^*({Q})$.
  3. For each corner point *q* of ${Q}$, perform a query in $\mathcal {D}_4$ to report all rectangles in $\mathcal {O}$ that contain *q*, and put them into $\mathcal {O}^*({Q})$.

The following lemma proves the correctness of our query procedure.

#### Lemma 1

Let $r_i,r_j$ be two rectangles in $\mathcal {O}$ such that $(r_i\cap r_j) \cap {Q}\ne \emptyset$. Then at least one of $r_i,r_j$ is put into $\mathcal {O}^*({Q})$ by the above query procedure.

**Fig. 2:**
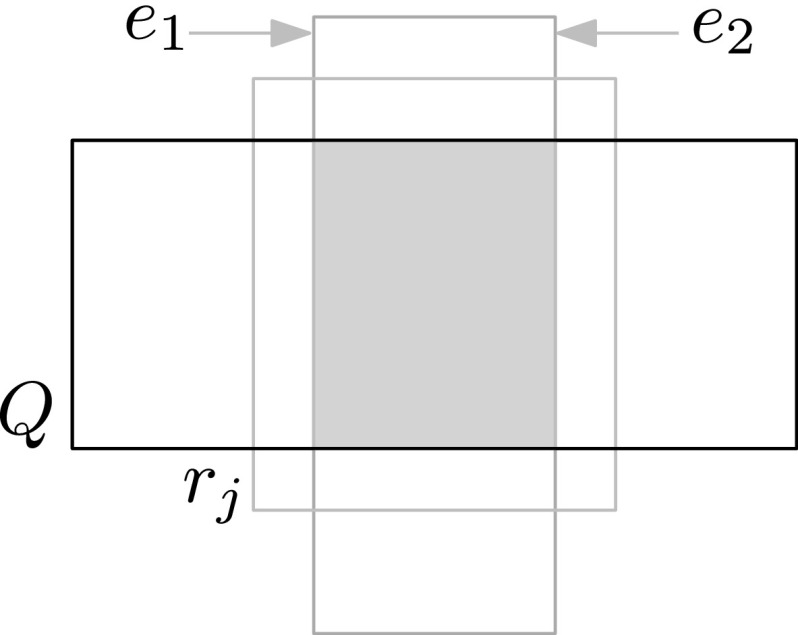
Example of Case B-3-I

#### Proof

Let $I:=(r_i\cap r_j) \cap {Q}$. Each edge of *I* is either contributed by $r_i$ or $r_j$, or by ${Q}$. Let *E*(*I*) denote the (possibly empty) set of edges of $r_i$ and $r_j$ that contribute an edge to *I*. We distinguish two cases, with various subcases.

Case A: At least one edge $e\in E(I)$ has an endpoint, *v*, inside ${Q}$. Now the witness sub-edge on *e* closest to *v* must intersect ${Q}$ and, hence, the corresponding rectangle will be put into $\mathcal {O}^*({Q})$ in Step 1(i).

Case B: All edges in *E*(*I*) cross ${Q}$ completely. We now have several subcases.

Case B-1: $|E(I)|\leqslant 1$. Now ${Q}$ contributes at least three edges to *I*, so at least one corner of *I* is a corner of ${Q}$. Hence, both $r_i$ and $r_j$ are put into $\mathcal {O}^*({Q})$ in Step 1(iii).

Case B-2: $|E(I)|\geqslant 3$. Since each edge of *E*(*I*) crosses ${Q}$ completely and $|E(I)|\geqslant 3$, both ${\textsf {V}}({Q})$ and $\textsf {H}({Q})$ are non-empty. Thus at least one of $r_i$ and $r_j$ is put into $\mathcal {O}^*({Q})$ in Step 2(ii).

Case B-3: $|E(I)|=2$. Let $e_1$ and $e_2$ denote the segments in *E*(*I*). If one of $e_1,e_2$ is vertical and the other is horizontal, we can use the argument from Case B-2. It remains to handle the case where $e_1$ and $e_2$ have the same orientation, say vertical.

Case B-3-i: Edges $e_1$ and $e_2$ belong to the same rectangle, say $r_i$, as in Fig. 2. If $e_1$ has an endpoint, *v*, inside $r_j$, then $e_1$ has a witness sub-edge starting at *v* that intersects ${Q}$, so $r_i$ is put into $\mathcal {O}^*({Q})$ in Step 1(i). If $r_j$ contains a corner of ${Q}$ then $r_j$ will be put into $\mathcal {O}^*({Q})$ in Step 1(iii). In the remaining case the right edge of $r_j$ crosses ${Q}$ and there are vertical edges completely crossing $r_j$ (namely $e_1$ and $e_2$). Hence, the rightmost edge completely crossing $r_j$, which is a witness for $r_j$, intersects ${Q}$. Thus $r_j$ is put into $\mathcal {O}^*({Q})$ in Step 1(i).

Case B-3-ii: Edge $e_1$ is an edge of $r_i$ and $e_2$ is an edge of $r_j$ (or vice versa). Assume without loss of generality that the *y*-coordinate of the top endpoint of $e_1$ is less than or equal to the *y*-coordinate of the top endpoint of $e_2$. Then the top endpoint, *v*, of $e_1$ must lie in $r_j$, and so $e_1$ has a witness sub-edge starting at *v* that intersects ${Q}$. Hence, $r_i$ is put into $\mathcal {O}^*({Q})$ in Step 1(i). $\square$

In the second part of the query procedure we need to report, for each rectangle $r_i$ in the seed set $\mathcal {O}^*({Q})$, the rectangles $r_j\in \mathcal {O}$ intersecting $r_i\cap {Q}$. Thus we store $\mathcal {O}$ in a data structure $\mathcal {D}_5$ that can report all rectangles intersecting a query rectangle. Putting everything together we obtain the following theorem.

#### Theorem 1

Let $\mathcal {O}$ be a set of *n* axis-aligned rectangles in ${\mathbb R}^2$. There is a data structure that uses $O(n\log n)$ storage and can report, for any axis-aligned query rectangle ${Q}$, all pairs of rectangles $r_i,r_j$ in $\mathcal {O}$ such that $r_i$ intersects $r_j$ inside ${Q}$ in $O((k+1)\log n)$ time, where *k* denotes the number of answers.

#### Proof

For the data structure $\mathcal {D}_1$ on the set *W* we use the data structure developed by Edelsbrunner et al. [13], which uses $O(n\log n)$ preprocessing time and storage, and has $O(\log n + \#\text{ answers })$ query time. For data structure $\mathcal {D}_2$ (and, similarly, $\mathcal {D}_3$) we note that a vertical segment $s_i := x_i \times [y_i,y'_i]$ crosses ${Q}:= [x_{{Q}},x'_{{Q}}]\times [y_{{Q}},y'_{{Q}}]$ if and only if the point $(x_i,y_i,y'_i)$ lies in the range $[x_{{Q}},x'_{{Q}}]\times [-\infty ,y_{{Q}}]\times [y'_{{Q}},\infty ]$. Hence, we can use the data structure of Afshani et al. [2], which uses $O(n\log n/\log \log n)$ storage and has $O(\log n + \#\text{ answers })$ query time. For data structure $\mathcal {D}_4$ we use the point-enclosure data structure developed by Chazelle [6], which uses *O*(*n*) storage and can be used to report all rectangles in $\mathcal {O}$ containing a query point in $O(\log n +\# \text{ answers })$ time.

Note that $|\mathcal {O}^*({Q})|\leqslant 2k+4$ where *k* is the total number of reported pairs. Indeed, each rectangle in $\mathcal {O}^*({Q})$ intersects at least one other rectangle inside ${Q}$ and for every reported pair we put at most two rectangles into the seed set; the extra term “+4” is because in Step 1 (iii) we may report at most one rectangle per corner of ${Q}$ that does not have an intersection inside ${Q}$. Hence, the time for Step 1 is $O(\log n + |\mathcal {O}^*({Q})|) = O(\log n +k)$.

It remains to analyze Step 2 of the query procedure, where we need to find for a given $r_i \in \mathcal {O}^*({Q})$ all $r_j\in \mathcal {O}$ such that $r_i\cap {Q}$ intersects $r_j$. First notice that a rectangle $r_j$ intersects a rectangle $r'_i := r_i\cap Q$ if and only if (i) a corner of $r_j$ is inside $r'_i$, or (ii) a corner of $r'_i$ is inside $r_j$, or (iii) an edge of $r_j$ intersects an edge of $r'_i$. Thus $\mathcal {D}_5$ consists of three components: All $r_j$ satisfying (i) can be found in $O(\log n +\# \text{ answers })$ time using a range tree with fractional cascading [11], which uses $O(n\log n)$ storage. All $r_j$ satisfying (ii) and (iii) can be found using, respectively, the data structure by Chazelle [6] and the one by Edelsbrunner et al. [13]. Thus the running time of Step 2 is $\sum _{r_i\in \mathcal {O}^*({Q})} O(\log n + k_i)$, where $k_i$ denotes the number of rectangles in $\mathcal {O}$ that intersect $r_i$ inside ${Q}$, and so the total time for Step 2 is $O((k+1)\log n)$. $\square$

### The 3-Dimensional Case

We now study the case where the set $\mathcal {O}$ of objects and the query range ${Q}$ are axis-aligned boxes in ${\mathbb R}^3$. We first present a solution for the general case, and then an improved solution for the special case where the input as well as the query are cubes. Both solutions use the same query strategy as above: we first find a seed set $\mathcal {O}^*({Q})$ that contains at least one object $o_i$ from every pair that intersects inside ${Q}$ and then we find all other objects intersecting $o_i$ inside ${Q}$.

**The general case** Let $\mathcal {O}:= \{b_1,\ldots ,b_n\}$ be a set of axis-aligned boxes. The pairs of boxes $b_i,b_j$ intersecting inside ${Q}$ come in three types: (i) $b_i\cap b_j$ fully contains ${Q}$, (ii) $b_i\cap b_j$ lies completely inside ${Q}$, (iii) $b_i\cap b_j$ intersects a face of ${Q}$.

Type (i) is easy to handle without using seed sets: we simply store $\mathcal {O}$ in a data structure for 3-dimensional point-enclosure queries [19], which allows us to report all boxes $b_i\in \mathcal {O}$ containing a query point in $O(\log ^2 n\cdot \log \log n+\#\text{ answers })$ time. If we query this structure with a corner *q* of ${Q}$ and report all pairs of boxes containing *q* then we have found all intersecting pairs of Type (i).

#### Lemma 2

We can find all intersecting pairs of boxes of Type (i) in $O(\log ^2 n\cdot \log \log n+k)$ time, where *k* is the number of such pairs, with a structure of size $O(n\log ^* n)$.

#### Remark

The query bound in Lemma 2 can be improved to $O(\log ^2 n+k)$ at the cost of $O(n\log n)$ storage, by using the data structure of Afshani et al. [3] instead of that of Rahul [19].

For Type (ii) we proceed as follows. Note that a vertex of $b_i\cap b_j$ is either a vertex of $b_i$ or $b_j$, or it is the intersection of an edge *e* of one of these two boxes and a face *f* of the other box. To handle the first case we create a set *W* of witness points, which contains for each box $b_i$ all its vertices that are contained in at least one other box. We store *W* in a data structure for 3-dimensional orthogonal range reporting [3]. In the query phase we then query this data structure with ${Q}$, and put all boxes corresponding to the witness vertices inside ${Q}$ into the seed set $\mathcal {O}^*({Q})$. For the second case we show next how to find the intersecting pairs *e*, *f* where *e* is a vertical edge (that is, parallel to the *z*-axis) and *f* is a horizontal face (that is, parallel to the *xy*-plane); the intersecting pairs with other orientations can be found in a similar way.

Let *E* be the set of vertical edges of the boxes in $\mathcal {O}$ and let *F* be the set of horizontal faces. We sort *F* by *z*-coordinate—we assume for simplicity that all *z*-coordinates of the faces are distinct—and partition *F* into $O(\sqrt{n})$ *clusters*: the cluster $F_1$ contains the first $\sqrt{n}$ faces in the sorted order, the second cluster $F_2$ contains the next $\sqrt{n}$ faces, and so on. We call the range between the minimum and maximum *z*-coordinate in a cluster its *z-range*. For each cluster $F_i$ we store, besides its *z*-range and the set $F_i$ itself, the following information. Let $E_i\subseteq E$ be the subset of edges that intersect at least one face in $F_i$, and let $\overline{E_i}$ denote the set of points obtained by projecting the edges in $E_i$ onto the *xy*-plane. We store $\overline{E_i}$ in a data structure $\mathcal {D}(\overline{E_i})$ for 2-dimensional orthogonal range reporting. Note that for a query box ${Q}$ whose *z*-range contains the *z*-range of $F_i$ we have: an edge $e\in E$ intersects at least one face $f\in F_i$ inside ${Q}$ if and only if $e\in E_i$ and $\overline{e}$ lies in $\overline{{Q}}$, the projection of ${Q}$ onto the *xy*-plane.

A query with a box ${Q}=[x_1:x_2]\times [y_1:y_2]\times [z_1:z_2]$ is now answered as follows. We first find the clusters $F_i$ and $F_j$ whose *z*-range contains $z_1$ and $z_2$, respectively, and we put (the boxes corresponding to) the faces in these clusters into the seed set $\mathcal {O}^*({Q})$. Next we perform, for each $i<t<j$, a query with the projected range $\overline{{Q}}$ in the data structure $\mathcal {D}(\overline{E_t})$. For each of the reported points $\overline{e}$ we put the box corresponding to the edge *e* into the seed set $\mathcal {O}^*({Q})$. Finally, we remove any duplicates from the seed set. This leads to the following lemma.

#### Lemma 3

Using a data structure of size $O(n\sqrt{n}\log ^\varepsilon n)$ we can find in time $O(\sqrt{n}\log n + k)$ a seed set $\mathcal {O}^*({Q})$ of $O(\sqrt{n}+k)$ boxes containing at least one box from every intersecting pair of Type (ii), where *k* is the number of such pairs. Here $\varepsilon >0$ is an arbitrary small, but fixed, positive constant.

#### Proof

The Type (ii) intersections $b_i \cap b_j$ either have a vertex that is a vertex of $b_i$ or $b_j$ inside ${Q}$, or they have an edge-face pair intersecting inside ${Q}$. To find seed objects for the former pairs we used $O(n(\log n/\log \log n)^2)$ storage and $O(\log n + k)$ query time [3], and we put *O*(*k*) boxes into the seed set. For the latter pairs, we used an approach based on clusters. For each cluster $F_i$ we have a data structure $\mathcal {D}(\overline{E_i})$, namely the 2-dimensional orthogonal range reporting structure of Chazelle [7], that uses $O(n\log ^\varepsilon n)$ storage, giving $O(n\sqrt{n}\log ^\varepsilon n)$ storage in total. Besides the $O(\sqrt{n})$ boxes in the two clusters $F_i$ and $F_j$, we put boxes into the seed set for the clusters $F_t$ with $i<t<j$, namely when querying the data structures $\mathcal {D}(\overline{E_t})$. This means that the same box may be put into $\mathcal {O}^*({Q})$ up to $\sqrt{n}$ times. (Note that these duplicates are later removed.) However, each copy we put into the seed set for some $F_t$ corresponds to a different intersecting pair. Together with the fact that the query time in each $\mathcal {D}(\overline{E_t})$ is $O(\log n +\# \text{ answers })$ this means the total query time and size of the seed set are as claimed. $\square$

It remains to handle the Type (iii) pairs, in which $b_i\cap b_j$ intersects a face of ${Q}$. We describe how to find the pairs such that $b_i\cap b_j$ intersects the bottom face of ${Q}$; the pairs intersecting the other faces can be found in a similar way.

We first sort the *z*-coordinates of the horizontal faces of the boxes in $\mathcal {O}$. For $1\leqslant i\leqslant 2\sqrt{n}$, let $h_i$ be a horizontal plane containing the $(i\sqrt{n})$th horizontal face. These planes partition ${\mathbb R}^3$ into $O(\sqrt{n})$ horizontal slabs $\Sigma _0,\ldots ,\Sigma _{2\sqrt{n}+1}$. We call a box $b\in \mathcal {O}$ *short* at $\Sigma _i$ if it has a horizontal face inside $\Sigma _i$, and we call it *long* if it completely crosses $\Sigma _i$. For each $\Sigma _i$, we store the short boxes in a list. We store the projections of the long boxes onto the *xy*-plane in a data structure $\mathcal {D}(\Sigma _i)$ for the 2-dimensional version of the problem, namely the structure of Theorem 1.

A query with the bottom face of ${Q}$ is now answered as follows. We first find the slab $\Sigma _i$ containing the face. We put all short boxes of $\Sigma _i$ into our seed set $\mathcal {O}^*({Q})$. We then perform a query with $\overline{{Q}}$, the projection of ${Q}$ onto the *xy*-plane, in the data structure $\mathcal {D}(\Sigma _i)$. For each answer we get from this 2-dimensional query—that is, each pair of projections intersecting inside $\overline{Q}$—we directly report the corresponding pair of long boxes. (There is no need to go through the seed set for these pairs.) This leads to the following lemma for the Type (iii) pairs.

#### Lemma 4

Using a data structure of size $O(n\sqrt{n}\log n)$ we can find in time $O(\sqrt{n}+k\log n)$ a seed set $\mathcal {O}^*({Q})$ of $O(\sqrt{n})$ boxes plus a collection $B({Q})$ of pairs of boxes intersecting inside ${Q}$ such that, for each pair of Type (iii) boxes, either at least one of these boxes is in $\mathcal {O}^*({Q})$ or $b_i,b_j$ is a pair in $B({Q})$.

In Step 2 of our query procedure we need to report all boxes $b_j\in \mathcal {O}$ intersecting a query box $B:={Q}\cap b_i$, where $b_i\in \mathcal {O}^*({Q})$. Note that *B* intersects $b_j$ if (i) *B* contains a vertex of $b_j$, or (ii) a vertex of *B* is contained in $b_j$, or (iii) an edge *e* of *B* intersects a face of $b_j$, or (iv) a face *f* of *B* intersects an edge of $b_j$. We build a data structure $\mathcal {D}^*$ consisting of several components to handle all of the cases.

All $r_j$ satisfying (i) and (ii) can be found using a 3-dimensional range reporting data structure and the 3-dimensional point-enclosure data structure of Afshani et al. [3]. Next we present the components of $\mathcal {D}^*$ needed to deal with (iii) and (iv).

For (iii), assume *e* is parallel to the *z*-axis and consider the faces of $b_j$ parallel to the *xy*-plane. Then we can use a 2-level structure whose first level is a tree on the *z*-coordinates of the faces, and whose second-level structures are 2-dimensional point-enclosure structures [6] on the projections onto the *xy*-plane. Note that *e* intersects a face *f* if and only if the *z*-coordinate of *f* lies in the *z*-range of *e*, and the projection of *e* onto the *xy*-plane lies inside the projection of *f* onto the *xy*-plane. A query with an edge *e* is now answered as follows. We first query the first level of tree with the *z*-range of *e* to locate $O(\log n)$ canonical nodes whose union covers the set of all faces whose *z*-coordinates lie in the queried range. We then query the associated structures of each of the selected nodes with the projection of *e* onto the *xy*-plane to report all faces that contain the point corresponding to the projected edge. Since the point-enclosure data structure uses $O(n\log n)$ storage and has $O(\log n)$ query time, this component of $\mathcal {D}^*$ needs $O(n\log ^2 n)$ storage and a query can be answered in $O(\log ^2 n + \# \text{ answers })$ time.

For (iv), we build a 2-level structure whose first level is a segment tree storing all the edges of all boxes. Each node $\nu$ of the first level is then associated with a 2D range tree storing the points corresponding to projections of the edges stored at the subtree rooted at $\nu$ onto the *xy*-plane. Now a query with a face *f* parallel to *xy*-plane can be answered as follows. We first query the first level of the structure with the *z*-coordinate of *f* to find a collection of $O(\log n)$ canonical nodes that together contain the set of edges whose *z*-ranges contain the queried *y*-coordinate. We then query the associated structures of each of the selected nodes with the projection of *f* onto the *xy*-plane to report all edges whose corresponding projections onto the *xy*-plane lie inside the queried projected range. Since this component of $\mathcal {D}^*$ needs $O(n\log ^2 n)$ storage and a query can be answered in only $O(\log ^2 n +\# \text{ answers })$ time we end up with the following theorem.

#### Theorem 2

Let $\mathcal {O}$ be a set of *n* axis-aligned boxes in ${\mathbb R}^3$. Then there is a data structure that uses $O(n\sqrt{n}\log n)$ storage and that allows us to report, for any axis-aligned query box ${Q}$, all pairs of boxes $b_i,b_j$ in $\mathcal {O}$ such that $b_i$ intersects $b_j$ inside ${Q}$ in $O((\sqrt{n} + k)\log ^2 n)$ time, where *k* denotes the number of answers.

As observed by Rahul [20] one can prove a conditional lower bound for our 3-dimensional queries by a reduction from set intersection queries. The set intersection query problem is to preprocess *m* sets $S_1,S_2,\ldots ,S_m$ of positive real numbers into a data structure that supports set intersection queries asking whether or not the sets $S_i$ and $S_j$ are disjoint, for given query indices *i* and *j*. Davoodi et al. [9] make the following conjecture. Here $\tilde{O}(\cdot )$ and $\tilde{\mathrm{\Omega }}(\cdot )$ hide polylog-factors.

#### Conjecture 1

Given a collection of *m* sets of *N* real numbers in total, where the maximum cardinality of the sets in polylogarithmic in *m*, any real-RAM data structure that supports set intersection queries in $\tilde{O}(t)$ time without using the floor function, requires $\tilde{\mathrm{\Omega }}((N/t)^2)$ storage, for $1\leqslant t\leqslant N$.

Davoodi et al. [9] use this conjecture for a conditional lower bound for diameter queries. As observed by Rahul [20], we can also use it to prove a conditional lower bound for our problem, as described next.

**Fig. 3:**
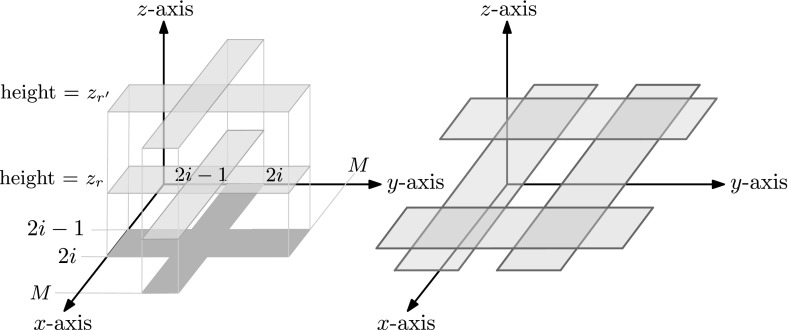
Left figure: the two boxes at height $z_r$ (resp. $z_{r'}$) are the boxes $b_1(i,z_r)$ and $b_2(i,z_r)$ (resp. $b_1(i,z_{r'}),b_2(i,z_{r'})$) for some integer $1\leqslant i\leqslant m$ and $z_r\in S_i$ (resp. $z_{r'}\in S_i$). Right figure: the two blue boxes are the boxes $b_1(i,z_r)$ and $b_2(i,z_r)$ for some integer $1\leqslant i\leqslant m$ and $z_r\in S_i$. The two red boxes are the boxes $b_1(j,z_r)$ and $b_2(j,z_r)$ for some integer $1\leqslant j\leqslant m$ with $j\ne i$ and $z_r\in S_j$. Either of the two red-blue intersections verifies the non-disjointness of $S_i$ and $S_j$ (Color figure online)

Let $S_1,S_2,\ldots ,S_m$ be a collection of sets and let $N=\sum _{i=1}^{m}|S_i|$. We transform the sets into a set of 2*N* boxes in ${\mathbb R}^3$. We map each element $z_r\in S_i$ into two boxes $b_1(i,z_r)$ and $b_2(i,z_r)$ as follows, letting $M:=2m+1$. We set $b_1(i,z_r):=[2i-1,2i]\times [0,M]\times z_r$, and we set $b_2(i,z_r):=[0,M]\times [2i-1,2i]\times z_r$. Note that the boxes of all elements of $S_i$ will have the same *xy*-projections. Only their *z*-ranges are different. See Fig. 3a for an example. In addition, notice that for $z_r\in S_i$ and $z_r\in S_j$ with $i\ne j$ the boxes $b_1(i,z_r)$ and $b_2(j,z_r)$ (as well as the boxes $b_1(j,z_r)$ and $b_2(i,z_r)$) intersect each other at $z=z_r$. Also, for $z_r\in S_i$ and $z'_r\in S_j$ with $z_r\ne z'_r$ none of the corresponding boxes of $S_i$ and $S_j$ intersect each other, since they have different *z*-ranges. Therefore, to verify the disjointness of $S_i$ and $S_j$, we ask to check if there is a pair of boxes that intersect each other inside the range $[2i-3/4,2i-1/4] \times [2j-3/4,2j-1/4] \times (-\infty ,+\infty )$. See Fig. 3b for an illustration.

The above reduction implies the following result.

#### Theorem 3

Suppose we have a data structure storing a set $\mathcal {O}$ of *n* axis-aligned boxes in ${\mathbb R}^3$ that uses *s*(*n*) storage and that can decide in *t*(*n*) time for a given query axis-aligned box ${Q}$ if there is a pair of boxes from $\mathcal {O}$ that intersect inside ${Q}$. Then we can build a data structure of size *s*(2*N*) supporting set intersection queries in *t*(2*N*) time, for input sets containing *N* elements in total.

Now Theorem 3 along with Conjecture 1 imply the following result.

#### Theorem 4

Let $\mathcal {O}$ be a set of *n* axis-aligned boxes in ${\mathbb R}^3$. Assuming Conjecture 1, any real-RAM data structure that can decide for a given query box ${Q}$ in $\tilde{O}(t)$ time, and without using the floor function, if there is a pair of boxes from $\mathcal {O}$ that intersect inside ${Q}$, requires $\tilde{\mathrm{\Omega }}((n/t)^2)$ storage.

*Fat boxes* Next we obtain better bounds when the boxes in $\mathcal {O}$ and the query box ${Q}$ are fat, that is, when their *aspect ratio*—the ratio between the length of the longest edge and the length of the shortest edge—is bounded by a constant $\alpha$. First we consider the case of cubes.

Let $\mathcal {O}:= \{c_1, \ldots , c_n\}$ be a set of *n* cubes in ${\mathbb R}^3$ and let ${Q}$ be the query cube. We compute a set *W* of witness points for each cube $c_i$, as follows. Let *e* be an edge of $c_i$, and consider the set $S(e) := e \cap \left( \cup _{j\ne i} c_j\right)$, that is, the part of *e* covered by the other cubes. We put the two extreme points from *S*(*e*)—in other words, the two points closest to the endpoints of *e*—into *W*. Similarly, we assign each face *f* of $c_i$ at most four witness points, namely points from $S(f) := f\cap (\cup _{j\ne i} c_j)$ that are extreme in the axis-aligned directions parallel to *f*. For example, if *f* is parallel to the *xy*-plane, then we take points of maximum and minimum *x*-coordinate in *S*(*f*) and points of maximum and minimum *y*-coordinate in *S*(*f*) as witnesses. Our data structure to find the seed set $\mathcal {O}^*({Q})$ now consists of the following components.

- We store the set *W* of witness points in a data structure $\mathcal {D}_1$ for 3-dimensional orthogonal range queries.
- We store $\mathcal {O}$ in a data structure $\mathcal {D}_2$ that allows us to report the set of cubes that contain a query point *q*.

The first step of the query procedure, where we compute $\mathcal {O}^*({Q})$, now proceeds as follows.

1. Perform a query in $\mathcal {D}_1$ to find all witness points inside ${Q}$. For each reported witness point, insert the corresponding cube into $\mathcal {O}^*({Q})$.
2. For each corner point *q* of ${Q}$, perform a query in $\mathcal {D}_2$ to report all cubes in $\mathcal {O}$ that contain *q*, and put them into $\mathcal {O}^*({Q})$.

The next lemma proves correctness of this procedure.

#### Lemma 5

Let $c_i,c_j$ be two cubes in $\mathcal {O}$ such that $(c_i\cap c_j) \cap {Q}\ne \emptyset$. Then at least one of $c_i,c_j$ is put into $\mathcal {O}^*({Q})$ by the above query procedure.

#### Proof

Suppose $c_i\cap c_j$ intersects ${Q}$, and assume without loss of generality that $c_i$ is not larger than $c_j$. If $c_i$ or $c_j$ contains a corner *q* of ${Q}$ then the corresponding cube will be put into the seed set when we perform a point-enclosure query with *q*, so assume $c_i$ and $c_j$ do not contain a corner. We have two cases.

Case A: $c_i$ does not intersect any edge of ${Q}$. Because $c_i$ and ${Q}$ are cubes, this implies that $c_i$ is contained in ${Q}$ or $c_i$ intersects exactly one face of ${Q}$. Assume that $c_i$ intersects the bottom face of ${Q}$; the cases where $c_i$ intersects another face and where $c_i$ is contained in ${Q}$ can be handled similarly. We claim that at least one of the vertical faces of $c_i$ contributes a witness point inside ${Q}$. To see this, observe that $c_j$ will intersect at least one vertical face, *f*, of $c_i$ inside ${Q}$, since $c_j$ intersects $c_i$ inside ${Q}$ and $c_i$ is not larger than $c_j$. Hence, the witness point on *f* with maximum *z*-coordinate will be inside ${Q}$. Thus $c_i$ will be put into $\mathcal {O}^*({Q})$.

**Fig. 4:**
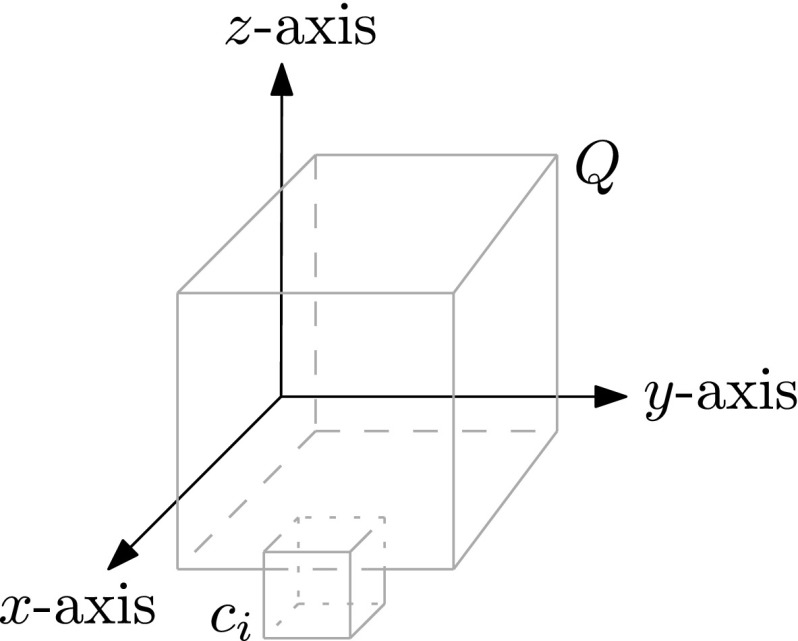
Case B in the proof of Lemma 5; $c_j$ is not shown

**Fig. 5:**
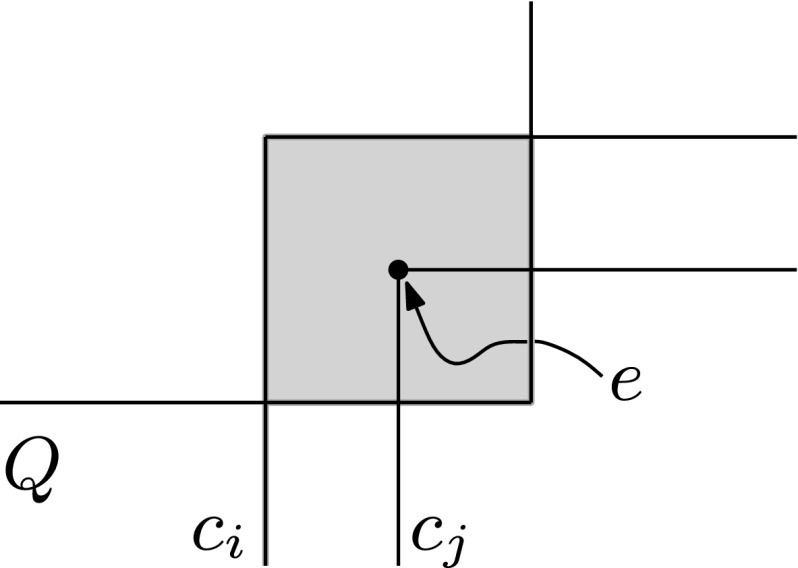
Cross-section of ${Q}$, $c_i$, and $c_j$ with a plane parallel to the *xz*-plane. The gray area indicates ${Q}\cap c_i$ in the cross-section

Case B: $c_i$ intersects one edge of ${Q}$. (If $c_i$ intersects more than one edge of ${Q}$ then it would contain a corner of ${Q}$.) Assume without loss of generality that $c_i$ intersects the bottom edge of the front face of ${Q}$; see Fig. 4. Observe that if $c_j$ intersects the top face of $c_i$ then the witness point of the face with minimum *x*-coordinate is inside ${Q}$. Similarly, if $c_j$ intersects the back face of $c_i$ (the face parallel to the *yz*-plane and with minimum *x*-coordinate) then the witness point of the face with maximum *z*-coordinate is inside ${Q}$. Otherwise, as illustrated in Fig. 5, $c_j$ must have an edge *e* parallel to the *y*-axis that intersects $c_i$ inside ${Q}$, and one of the witness points on *e* will be inside ${Q}$—note that *e* lies fully inside ${Q}$ because $c_j$ does not contain a corner of ${Q}$. $\square$

To handle fat boxes, we need the following observation.

#### Observation 1

Let *b* be a box of aspect ratio $\alpha$. Then we can cover *b* by $O(\alpha ^2)$ cubes such that any cube in the covering intersects at most three other cubes from the covering.

To adapt the above solution to boxes of aspect ratio at most $\alpha$, we cover each box $b_i\in \mathcal {O}$ by $O(\alpha ^2)$ cubes, and preprocess the resulting collection $\widetilde{\mathcal {O}}$ of cubes as described above, making sure we do not introduce witness points for pairs of cubes used in the covering of the same box $b_i$. To perform a query, we cover ${Q}$ by $O(\alpha ^2)$ query cubes and compute a seed set for each query cube. We take the union of these seed sets, replace the cubes from $\widetilde{\mathcal {O}}$ in the seed set by the corresponding boxes in $\mathcal {O}$, and filter out duplicates. This gives us our seed set $\mathcal {O}^*({Q})$ for the second phase of the query procedure.

In the second phase we take each $b_i\in \mathcal {O}^*({Q})$ and report all $b_j\in \mathcal {O}$ intersecting $b_i\cap {Q}$, using the data structure $\mathcal {D}^*$ described just before Theorem 2. We obtain the following theorem.

#### Theorem 5

Let $\mathcal {O}$ be a set of *n* axis-aligned boxes in ${\mathbb R}^3$ of aspect ratio at most $\alpha$. Then there is a data structure that uses $O(\alpha ^2 n\log ^2 n)$ storage and that allows us to report, for any axis-aligned query box ${Q}$ of aspect ratio at most $\alpha$, all pairs of cubes $c_i,c_j$ in $\mathcal {O}$ such that $c_i$ intersects $c_j$ inside ${Q}$ in $O(\alpha ^2(k+1)\log ^2 n)$ time, where *k* denotes the number of answers.

#### Proof

The data structures $\mathcal {D}_1$ and $\mathcal {D}_2$ can be implemented such that they use $O(n(\log n/\log \log n)^2)$ storage in total, and have $O(\log n + \#\text{ answers })$ and $O(\log ^2 n/\log \log n + \#\text{ answers })$ query time, respectively [3]. Since Step 2 of the query procedure is the same as the second step of query procedure of Sect. 2.2 we can use the data structures that we designed there, which need $O(n\log ^2 n)$ storage and have $O(\log ^2 n + \#\text{ answers })$ query time. The conversion of boxes of aspect ratio $\alpha$ to cubes give an additional factor $O(\alpha ^2)$. Each input box now has $O(\alpha ^2)$ witness points, but each witness point will be reported by at most three of the query cubes, by Observation 1. Similarly, each corner of a query cube is inside at most two cubes from the covering of any box $b_i\in \mathcal {O}$. $\square$

## Objects with Small Union Complexity in ${\mathbb R}^2$

In the previous section we presented efficient solutions for the case where $\mathcal {O}$ consists of axis-aligned rectangles. In this section we obtain results for classes of constant-complexity objects (which may have curved boundaries) with small union complexity. More precisely, we need that *U*(*n*), the maximum union complexity of any set of *n* objects from the class, is small. This is for instance the case for disks (where $U(m)=O(m)$ [18]) and for locally fat objects (where $U(m)=m2^{O(\log ^* m)}$ [5]).

In Step 2 of the query algorithm of the previous section, we performed a range query with $o_i\cap {Q}$ for each $o_i\in \mathcal {O}^*({Q})$. When we are dealing with arbitrary objects, this will be expensive, so we modify our query procedure.

1. Compute a seed set $\mathcal {O}^*({Q})\subseteq \mathcal {O}$ of objects such that, for any two objects $o_i,o_j$ in $\mathcal {O}$ intersecting inside ${Q}$, both $o_i$ and $o_j$ are in $\mathcal {O}^*({Q})$. (Contrary to before, where we only required one of $o_i,o_j$ to be in the seed set.)
2. Compute all intersecting pairs of objects in the set $\{ o_i\cap {Q}: o_i \in \mathcal {O}^*({Q}) \}$ by a plane-sweep algorithm.

**Fig. 6:**
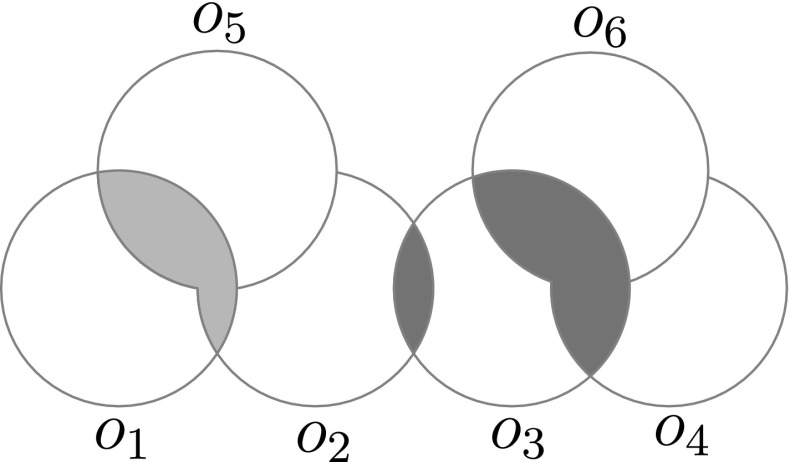
An illustration of the regions $o_i^*$ for disks. Only $o_1^*$ and $o_3^*$ are shown. $o_1^*$ is shown in red, and $o_3^*$ is shown in blue (Color figure online)

Next we describe how to efficiently find $\mathcal {O}^*({Q})$, which should contain all objects intersecting at least one other object inside ${Q}$, when the union complexity *U*(*n*) is small. For each object $o_i\in \mathcal {O}$ we define $o_i^* := \bigcup _{{o_j\in \mathcal {O}},{j \ne i}} (o_i \cap o_j)$ as the union of all intersections between $o_i$ and all other objects in $\mathcal {O}$. See Fig. 6 for an illustration. Let $|o_i^*|$ denote the complexity (that is, number of vertices and edges) of $o_i^*$.

### Lemma 6

$$\sum _{i=1}^n |o_i^*| = O(U(n))$$.

### Proof

Consider the arrangement induced by the objects in $\mathcal {O}$. We define the *level* of a vertex *v* in this arrangement as the number of objects from $\mathcal {O}$ that contain *v* in their interior. We claim that every vertex of any $o_i^*$ is a level-0 or level-1 vertex. Indeed, a level-*k* vertex for $k>1$ is in the interior of more than one object, which implies it cannot be a vertex of any $o_i^*$.

Since the level-0 vertices are exactly the vertices of the union of $\mathcal {O}$, the total number of level-0 vertices is *U*(*n*). It follows from the Clarkson–Shor technique [8] that the number of level-1 vertices is *O*(*U*(*n*)) as well. The lemma now follows, because each level-0 or level-1 vertex contributes to at most two different $o_i^*$’s. $\square$

Our goal in Step 1 is to find all objects $o_i$ such that $o_i^*$ intersects ${Q}$. To this end consider the connected components of $o_i^*$. If $o_i^*$ intersects ${Q}$ then one of these components lies completely inside ${Q}$ or an edge of ${Q}$ intersects $o_i^*$.

### Lemma 7

We can find all $o_i^*$ that have a component completely inside ${Q}$ in $O(\log n + k)$ time, where *k* is the number of pairs of objects that intersect inside ${Q}$, with a data structure that uses $O(U(n)\log n)$ storage.

### Proof

For each $o_i$, take an arbitrary representative point inside each component of $o_i^*$, and store all the representative points in a structure for orthogonal range reporting. By Lemma 6 we store *O*(*U*(*n*)) points, and so the structure for orthogonal range reporting uses $O(U(n)\log n)$ storage.

The query time is $O(\log n + t)$, where *t* is the number of representative points inside ${Q}$. This implies the query time is $O(\log n + k)$, because if $o_i^*$ has $t_i$ representative points inside ${Q}$ then $o_i$ intersects $\mathrm{\Omega }(t_i)$ other objects inside ${Q}$. This is true because the objects have constant complexity, so a single object $o_j$ cannot generate more than a constant number of components of $o_i^*$. $\square$

Next we describe a data structure for reporting all $o_i^*$ intersecting a vertical edge of ${Q}$; the horizontal edges of ${Q}$ can be handled similarly. The data structure is a balanced binary tree $\mathcal {T}$, whose leaves are in one-to-one correspondence to the objects in $\mathcal {O}$. For an (internal or leaf) node $\nu$ in $\mathcal {T}$, let $\mathcal {T}(\nu )$ denote the subtree rooted at $\nu$ and let $\mathcal {O}(\nu )$ denote the set of objects corresponding to the leaves of $\mathcal {T}(\nu )$. Define $\mathcal {U}(\nu ) := \cup _{o_i \in \mathcal {O}(\nu )} o_i^*$. At node $\nu$, we store a point-location data structure [12] on the trapezoidal map of $\mathcal {U}(\nu )$. (If the objects are curved, then the “trapezoids” may have curved top and bottom edges.)

### Lemma 8

The tree $\mathcal {T}$ uses $O(U(n)\log n)$ storage and allows us to report all $o_i^*$ intersecting a vertical edge *s* of ${Q}$ in $O((t+1)\log ^2 n)$ time, where *t* is the number of answers.

### Proof

To report all $o_i^*$ intersecting *s* we walk down $\mathcal {T}$, only visiting the nodes $\nu$ such that *s* intersects $\mathcal {U}(\nu )$. This way we end up in the leaves corresponding to the $o_i^*$ intersecting *s*. To decide if we have to visit a child $\nu$ of an already visited node, we do a point location with both endpoints of *s* in the trapezoidal map of $\mathcal {U}(\nu )$. Now *s* intersects $\mathcal {U}(\nu )$ if and only if one of these endpoints lies in a trapezoid inside $\mathcal {U}(\nu )$ and/or the two endpoints lie in different trapezoids. Thus we spend $O(\log n)$ time for the decision. Since we visit $O(t\log n)$ nodes, the total query time is as claimed.

To analyze the storage we claim that the sum of the complexities of $\mathcal {U}(\nu )$ over all nodes $\nu$ at any fixed height of $\mathcal {T}$ is *O*(*U*(*n*)). The bound on the storage then follows because the point-location data structures take linear space [12] and the height of $\mathcal {T}$ is $O(\log n)$. It remains to prove the claim. Consider a node $\nu$ at a given height *h* in $\mathcal {T}$. Lemma 9 argues that each vertex in $\mathcal {U}(\nu )$ is either a level-0 or level-1 vertex of the arrangement induced by the objects in $\mathcal {O}(\nu )$, or a vertex of $o^*_i$, for some $o_i$ in $\mathcal {O}(\nu )$. The proof of the claim then follows from the following two facts. First, the number of vertices of the former type is $O(U(|\mathcal {O}(\nu )|))$, which sums to *O*(*U*(*n*)) over all nodes at height *h*. Second, by Lemma 6 the number of vertices of the latter type over all nodes at height *h* sums to *O*(*U*(*n*)). $\square$

**Fig. 7:**
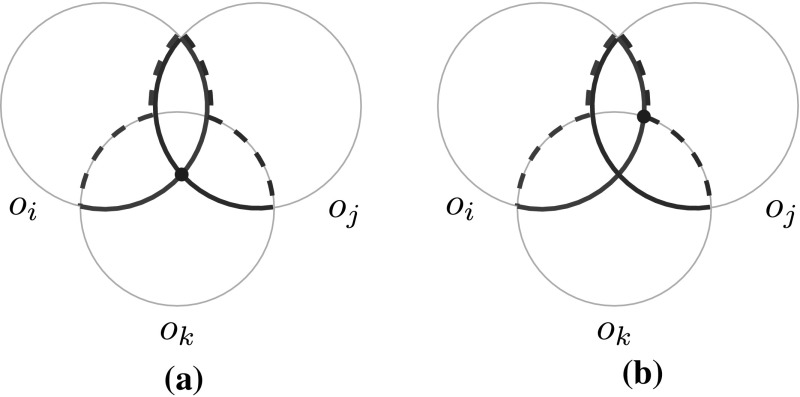
Different cases in the proof of Lemma 9. To simplify the presentation we assumed the objects are disks. $o^*_i$ and $o^*_j$ are surrounded by dark green and dark red, respectively. Regular arcs are in solid and irregular arcs are in dashed. The blue vertex refers to vertex *u* in the proof. **a** Case A in the Proof of Lemma 9. **b** Case B in the Proof of Lemma 9 (Color figure online)

### Lemma 9

Each vertex in $\mathcal {U}(\nu )$ is either a level-0 or level-1 vertex of the arrangement induced by the objects in $\mathcal {O}(\nu )$, or a vertex of $o^*_i$, for some $o_i$ in $\mathcal {O}(\nu )$.

### Proof

Define $\mathcal {O}^*(\nu ) := \{ o_i^* : o_i \in \mathcal {O}(\nu ) \}$. Any vertex *u* of $\mathcal {U}(\nu )$ that is not a vertex of some $o_i^* \in \mathcal {O}^*(\nu )$ must be an intersection of the boundaries of some $o_i^*,o_j^*\in \mathcal {O}(\nu )$. Note that the boundary $\partial o_i^*$ of an object $o_i^*$ consists of two types of pieces: *regular arcs*, which are parts of the boundary of $o_i$ itself, and *irregular arcs*, which are parts of the boundary of some other object $o_k$. To bound the number of vertices of $\mathcal {U}(\nu )$ of the form $\partial o_i^* \cap \partial o_j^*$ we now distinguish three cases.

Case A: Intersections between two regular arcs. In this case *u* is either a level-0 vertex of the arrangement defined by $\mathcal {O}(\nu )$ (namely when *u* is contained in no other object $o_k\in \mathcal {O}(\nu )$), or a level-1 vertex of that arrangement (when *u* is contained in a single object $o_k\in \mathcal {O}(\nu )$). Note that *u* cannot be contained in two objects from $\mathcal {O}(\nu )$, because then *u* would be in the interior of some $o_k^*\in \mathcal {O}^*(\nu )$, contradicting that *u* is a vertex of $\mathcal {U}(\nu )$. See Fig. 7a.

Case B: Intersections between a regular arc and an irregular arc. Without loss of generality, assume that *u* is the intersection of a regular arc of $\partial o_i^*$ and an irregular arc of $\partial o_j^*$. Note that this implies that *u* lies in the interior of $o_j$. If there is no other object $o_k\in \mathcal {O}$ containing *u* then *u* would be a vertex of $o_j^*$, and if there is at least one object $o_k\in \mathcal {O}$ containing *u* then *u* would not lie on $\partial o_j^*$. So, under the assumption that *u* is not already a vertex of $o_j^*$, Case B does not happen. See Fig. 7b.

Case C: Intersections between two irregular arcs. In this case *u* lies in the interior of both $o_i$ and $o_j$. But then *u* should also be in the interior of $o_i^*$ and $o_j^*$, so this case cannot happen. $\square$

Putting everything together we obtain the following result.

### Theorem 6

Let $\mathcal {O}$ be a set of *n* constant-complexity objects in ${\mathbb R}^2$ from a class of objects such that the maximum union complexity of any *m* objects from the class is *U*(*m*). Then there is a data structure that uses $O(U(n)\log n)$ storage and that allows us to report for any axis-aligned query rectangle ${Q}$, in $O((k+1)\log ^2 n)$ time all pairs of objects $o_i,o_j$ in $\mathcal {O}$ such that $o_i$ intersects $o_j$ inside ${Q}$, where *k* denotes the number of answers.

## Discussion

We presented data structures for finding intersecting pairs of objects inside a query rectangle. An obvious open problem is whether our bounds can be improved. In particular, one would hope that better solutions are possible for 3-dimensional boxes, where we obtained $O((k+\sqrt{n})\cdot {{\mathrm{polylog\,}}}n)$ query time with $O(n\sqrt{n}\log n)$ storage. (We can reduce the query time to $O((k+m)\cdot {{\mathrm{polylog\,}}}n)$, for any $1\leqslant m\leqslant \sqrt{n}$, but at the cost of increasing the storage to $O((n^2/m)\cdot {{\mathrm{polylog\,}}}n)$.)

Two settings where we have not been able to obtain efficient solutions are when the objects are balls in ${\mathbb R}^3$, and when they are arbitrary segments in ${\mathbb R}^2$. Especially the latter case is challenging. Indeed, suppose $\mathcal {O}$ consists of *n* / 2 horizontal lines and *n* / 2 lines of slope 1. Suppose furthermore that the query is a vertical line $\ell$ and that we only want to check if $\ell$ contains at least one intersection. A data structure for this can be used to solve the following 3Sum-hard problem: given three sets of parallel lines, decide if there is a triple intersection [14]. Thus it is unlikely that we can obtain a solution with sublinear query time and subquadratic preprocessing time. However, storage is not the same as preprocessing time. This raises the following question: is it possible to obtain sublinear query time with subquadratic storage? Another interesting question would be to see whether or not the query time in Theorem 1 can be improved to $O(\log n +k)$.

## Footnotes

1 Here $\log ^* n$ denotes the iterated logarithm.

## References

[1] Abam M, Carmi P, Farshi M, Smid M (2013). On the power of semi-separated pair decomposition. Comput. Geom..

[2] Afshani, P., Arge, L., Larsen, K.D.: Orthogonal range reporting in three and higher dimensions. In: Proceedings of IEEE Symposium, pp. 149–158 (2009)

[3] Afshani, P., Arge, L., Larsen, K.D.: Orthogonal range reporting: query lower bounds, optimal structures in 3-d, and higher-dimensional improvements. In: Proceedings of ACM Symposium on Computational Geometry, pp. 240–246 (2010)

[4] Agarwal PK, Erickson J (1999). Geometric range searching and its relatives. Contemp. Math..

[5] Aronov B, de Berg M, Ezra E, Sharir M (2014). Improved bounds for the union of locally fat objects in the plane. SIAM J. Comput..

[6] Chazelle B (1986). Filtering search: a new approach to query-answering. SIAM J. Comput..

[7] Chazelle B (1988). A functional approach to data structures and its use in multidimensional searching. SIAM J. Comput..

[8] Clarkson KL, Shor PW (1989). Applications of random sampling in computational geometry. II. Discrete Comput. Geom..

[9] Davoodi, P., Smid, M., van Walderveen, F.: Two-dimensional range diameter queries. In: Proceedings of Latin American Symposium on Theoretical Informatics, pp. 219-230 (2012)

[10] Das AS, Gupta P, Srinathan K (2011). Data structures for extension violations in a query range. J. Math. Model. Algorithms.

[11] de Berg M, Cheong O, van Kreveld M, Overmars M (2008). Computational Geometry: Algorithms and Applications.

[12] Edelsbrunner H, Guibas LJ, Stolfi J (1986). Optimal point location in a monotone subdivision. SIAM J. Comput..

[13] Edelsbrunner H, Overmars MH, Seidel R (1984). Some methods of computational geometry applied to computer graphics. Comput. Vis. Graphics Image Proc..

[14] Gajentaan A, Overmars MH (1995). On a class of $$O(n^2)$$ problems in computational geometry. Comput. Geom. Theory Appl..

[15] Goodman, J.E., O’Rourke, J.: Range searching. In: Handbook of Discrete and Computational Geometry, Chapter 36, 2nd edn (2004)

[16] Gupta P (2006). Range-aggregate query problems involving geometric aggregation operations. Nord. J. Comput..

[17] Gupta P, Janardan R, Kumar Y, Smid M (2014). Data structures for range-aggregate extent queries. Comput. Geom. Theory Appl..

[18] Keden K, Livne R, Pach J, Sharir M (1986). On the union of Jordan regions and collision-free translational motion amidst polygonal obstacles. Discr. Comput. Geom..

[19] Rahul, S.: Improved bounds for orthogonal point enclosure query and point location in orthogonal subdivisions in $${\mathbb{R}}^3$$. In: ACM-SIAM Symposium on Discrete Algorithms, pp. 200–211 (2015)

[20] Rahul, S.: Personal communication

[21] Rahul, S., Das, A.S., Rajan, K.S., Srinatan, K.: Range-aggregate queries involving geometric aggregation operations. In: Workshop on Algorithms and Computation, vol. 1, pp. 122–133 (2011)

